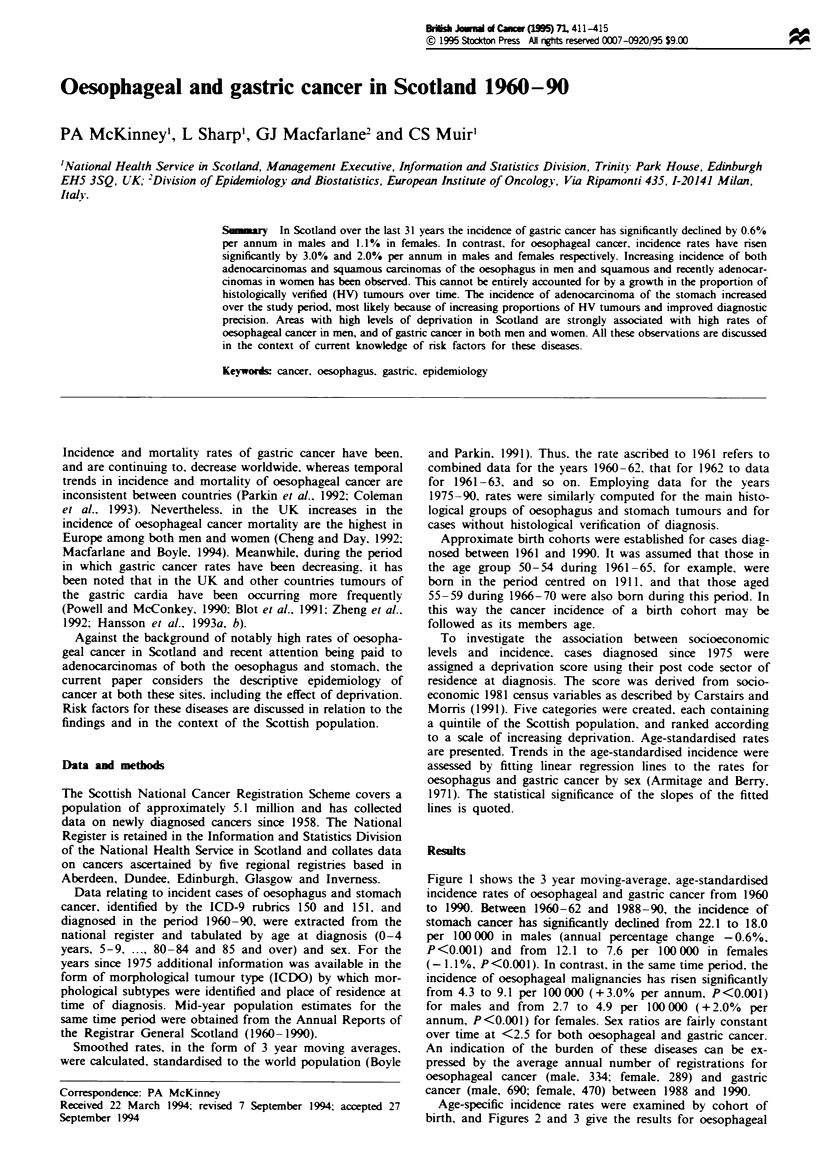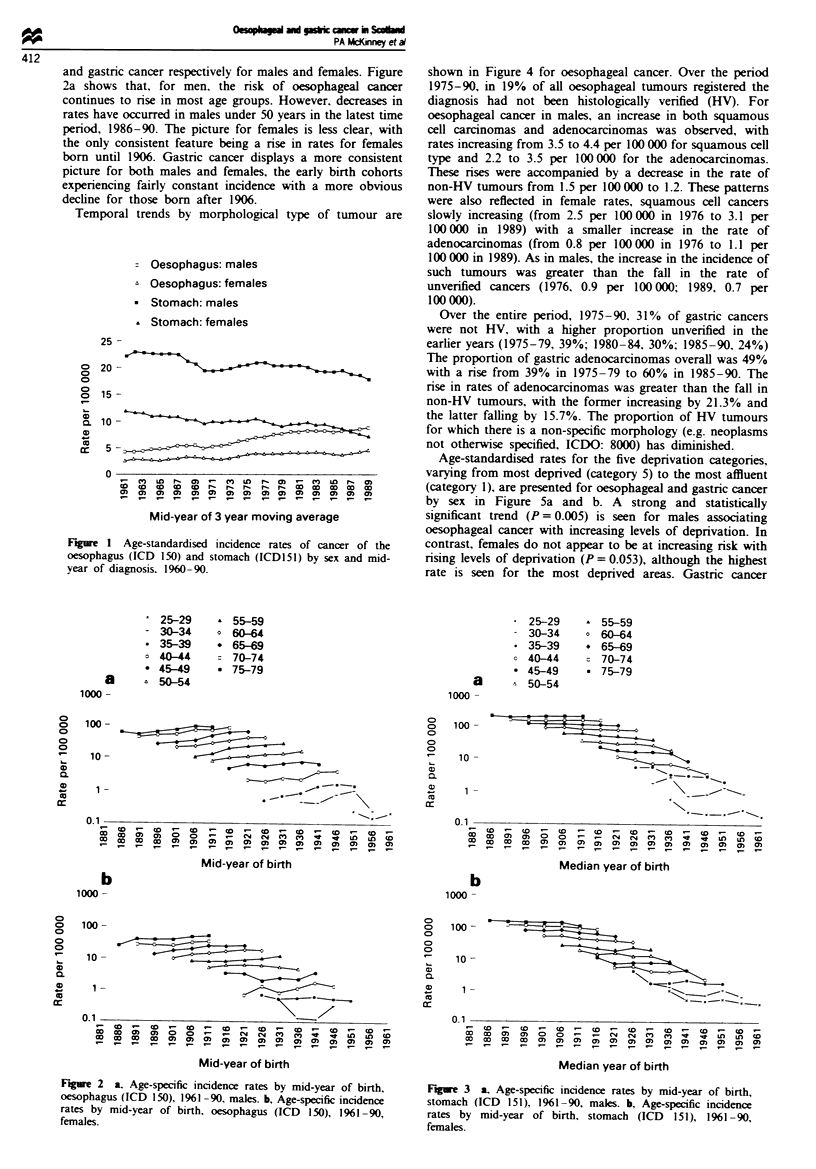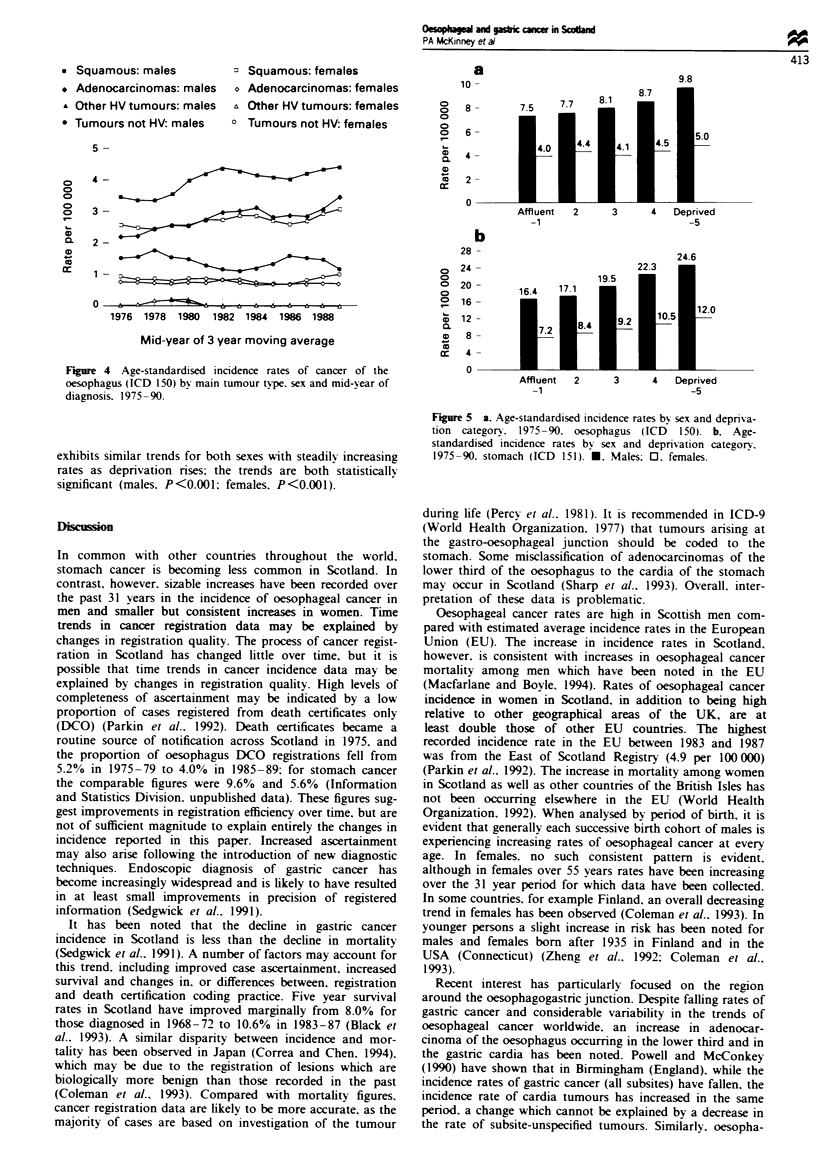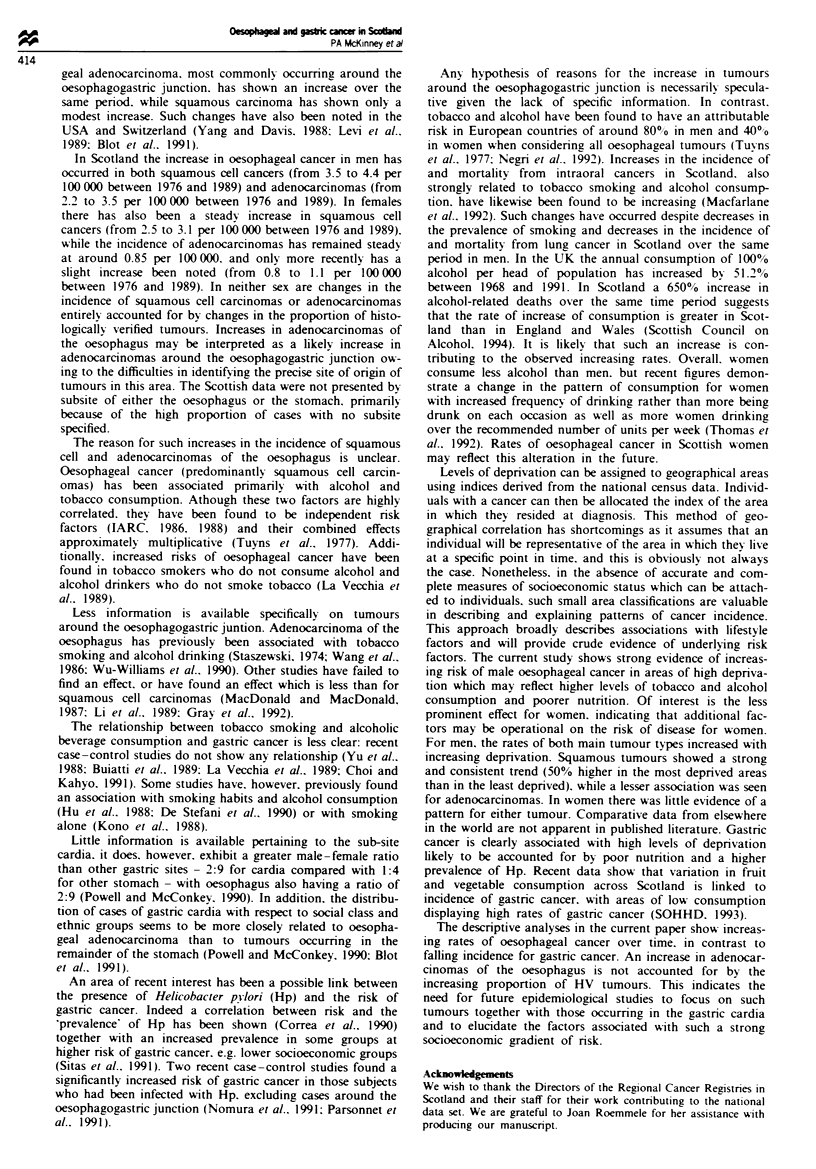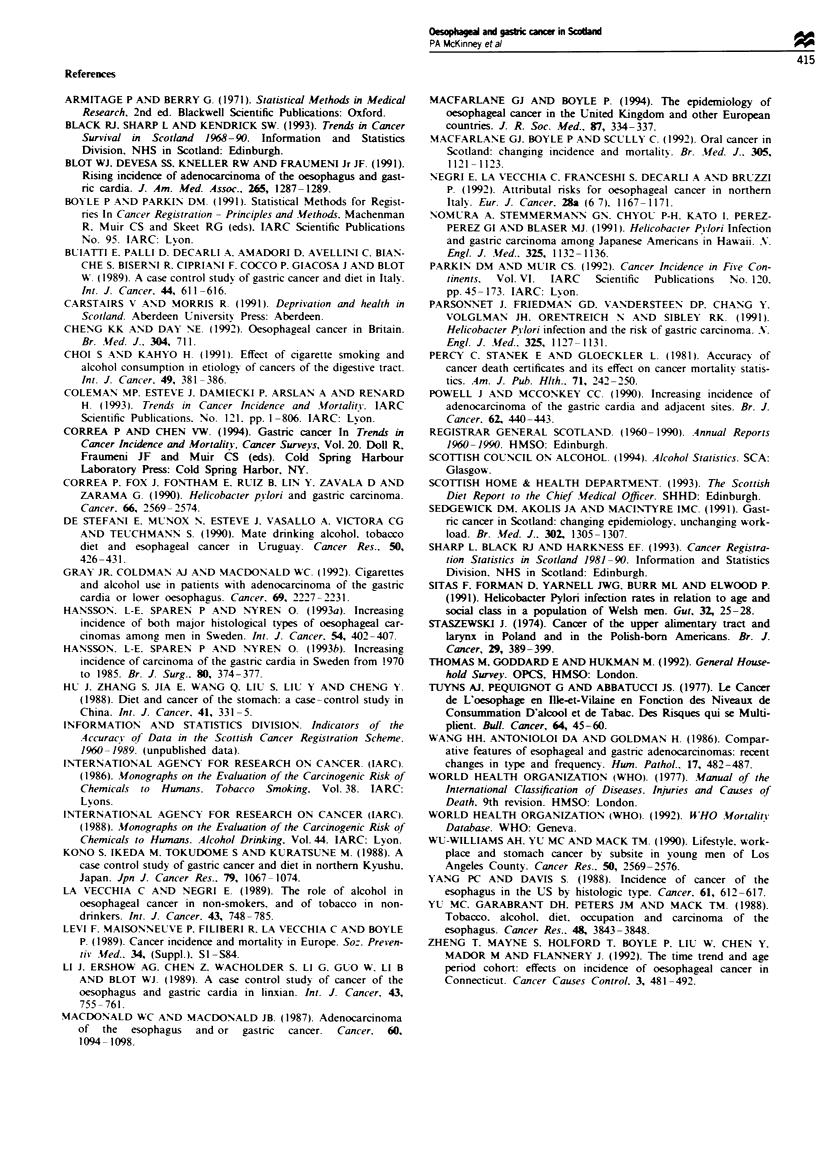# Oesophageal and gastric cancer in Scotland 1960-90.

**DOI:** 10.1038/bjc.1995.84

**Published:** 1995-02

**Authors:** A. McKinney, L. Sharp, G. J. Macfarlane, C. S. Muir

**Affiliations:** National Health Service in Scotland, Management Executive, Information and Statistics Division, Edinburgh, UK.

## Abstract

In Scotland over the last 31 years the incidence of gastric cancer has significantly declined by 0.6% per annum in males and 1.1% in females. In contrast, for oesophageal cancer, incidence rates have risen significantly by 3.0% and 2.0% per annum in males and females respectively. Increasing incidence of both adenocarcinomas and squamous carcinomas of the oesophagus in men and squamous and recently adenocarcinomas in women has been observed. This cannot be entirely accounted for by a growth in the proportion of histologically verified (HV) tumours over time. The incidence of adenocarcinoma of the stomach increased over the study period, most likely because of increasing proportions of HV tumours and improved diagnostic precision. Areas with high levels of deprivation in Scotland are strongly associated with high rates of oesophageal cancer in men, and of gastric cancer in both men and women. All these observations are discussed in the context of current knowledge of risk factors for these diseases.


© 1995 Stockton Press All rights reserved 0007-0920/95 $9.00

### Oesophageal and gastric cancer in Scotland 1960-90

PA McKinney[1], L Sharp[1], GJ Macfarlane[2] and CS Muir[1]

[1]*National Health Service in Scotland, Management Executive, Information and Statistics Division, Trinity Park House, Edinburgh EH5 3SQ, UK;* [2]*Division of Epidemiology and Biostatistics, European Institute of Oncology, Via Ripamonti 435, I-20141 Milan, Italy.*

**Summary** In Scotland over the last 31 years the incidence of gastric cancer has significantly declined by 0.6% per annum in males and 1.1% in females. In contrast, for oesophageal cancer, incidence rates have risen significantly by 3.0% and 2.0% per annum in males and females respectively. Increasing incidence of both adenocarcinomas and squamous carcinomas of the oesophagus in men and squamous and recently adenocarcinomas in women has been observed. This cannot be entirely accounted for by a growth in the proportion of histologically verified (HV) tumours over time. The incidence of adenocarcinoma of the stomach increased over the study period, most likely because of increasing proportions of HV tumours and improved diagnostic precision. Areas with high levels of deprivation in Scotland are strongly associated with high rates of oesophageal cancer in men, and of gastric cancer in both men and women. All these observations are discussed in the context of current knowledge of risk factors for these diseases.

**Keywords:** cancer, oesophagus, gastric, epidemiology

Incidence and mortality rates of gastric cancer have been, and are continuing to, decrease worldwide, whereas temporal trends in incidence and mortality of oesophageal cancer are inconsistent between countries (Parkin *et al*., 1992; Coleman *et al*., 1993). Nevertheless, in the UK increases in the incidence of oesophageal cancer mortality are the highest in Europe among both men and women (Cheng and Day, 1992; Macfarlane and Boyle, 1994). Meanwhile, during the period in which gastric cancer rates have been decreasing, it has been noted that in the UK and other countries tumours of the gastric cardia have been occurring more frequently (Powell and McConkey, 1990; Blot *et al*., 1991; Zheng *et al*., 1992; Hansson *et al*., 1993*a*, *b*).

Against the background of notably high rates of oesophageal cancer in Scotland and recent attention being paid to adenocarcinomas of both the oesophagus and stomach, the current paper considers the descriptive epidemiology of cancer at both these sites, including the effect of deprivation. Risk factors for these diseases are discussed in relation to the findings and in the context of the Scottish population.

### Data and methods

The Scottish National Cancer Registration Scheme covers a population of approximately 5.1 million and has collected data on newly diagnosed cancers since 1958. The National Register is retained in the Information and Statistics Division of the National Health Service in Scotland and collates data on cancers ascertained by five regional registries based in Aberdeen, Dundee, Edinburgh, Glasgow and Inverness.

Data relating to incident cases of oesophagus and stomach cancer, identified by the ICD-9 rubrics 150 and 151, and diagnosed in the period 1960-90, were extracted from the national register and tabulated by age at diagnosis (0-4 years, 5-9, ..., 80-84 and 85 and over) and sex. For the years since 1975 additional information was available in the form of morphological tumour type (ICDO) by which morphological subtypes were identified and place of residence at time of diagnosis. Mid-year population estimates for the same time period were obtained from the Annual Reports of the Registrar General Scotland (1960-1990).

Smoothed rates, in the form of 3 year moving averages, were calculated, standardised to the world population (Boyle and Parkin, 1991). Thus, the rate ascribed to 1961 refers to combined data for the years 1960-62, that for 1962 to data for 1961-63, and so on. Employing data for the years 1975-90, rates were similarly computed for the main histological groups of oesophagus and stomach tumours and for cases without histological verification of diagnosis.

Approximate birth cohorts were established for cases diagnosed between 1961 and 1990. It was assumed that those in the age group 50-54 during 1961-65, for example, were born in the period centred on 1911, and that those aged 55-59 during 1966-70 were also born during this period. In this way the cancer incidence of a birth cohort may be followed as its members age.

To investigate the association between socioeconomic levels and incidence, cases diagnosed since 1975 were assigned a deprivation score using their post code sector of residence at diagnosis. The score was derived from socioeconomic 1981 census variables as described by Carstairs and Morris (1991). Five categories were created, each containing a quintile of the Scottish population, and ranked according to a scale of increasing deprivation. Age-standardised rates are presented. Trends in the age-standardised incidence were assessed by fitting linear regression lines to the rates for oesophagus and gastric cancer by sex (Armitage and Berry, 1971). The statistical significance of the slopes of the fitted lines is quoted.

### Results

Figure 1 shows the 3 year moving-average, age-standardised incidence rates of oesophageal and gastric cancer from 1960 to 1990. Between 1960-62 and 1988-90, the incidence of stomach cancer has significantly declined from 22.1 to 18.0 per 100 000 in males (annual percentage change −0.6%, $P<0.001$) and from 12.1 to 7.6 per 100 000 in females (−1.1%, $P<0.001$). In contrast, in the same time period, the incidence of oesophageal malignancies has risen significantly from 4.3 to 9.1 per 100 000 (+3.0% per annum, $P<0.001$) for males and from 2.7 to 4.9 per 100 000 (+2.0% per annum, $P<0.001$) for females. Sex ratios are fairly constant over time at <2.5 for both oesophageal and gastric cancer. An indication of the burden of these diseases can be expressed by the average annual number of registrations for oesophageal cancer (male, 334; female, 289) and gastric cancer (male, 690; female, 470) between 1988 and 1990.

Age-specific incidence rates were examined by cohort of birth, and Figures 2 and 3 give the results for oesophageal

Correspondence: PA McKinney
Received 22 March 1994; revised 7 September 1994; accepted 27 September 1994

and gastric cancer respectively for males and females. Figure 2a shows that, for men, the risk of oesophageal cancer continues to rise in most age groups. However, decreases in rates have occurred in males under 50 years in the latest time period, 1986–90. The picture for females is less clear, with the only consistent feature being a rise in rates for females born until 1906. Gastric cancer displays a more consistent picture for both males and females, the early birth cohorts experiencing fairly constant incidence with a more obvious decline for those born after 1906.

Temporal trends by morphological type of tumour are

**Figure 1** Age-standardised incidence rates of cancer of the oesophagus (ICD 150) and stomach (ICD151) by sex and mid-year of diagnosis, 1960–90.

shown in Figure 4 for oesophageal cancer. Over the period 1975–90, in 19% of all oesophageal tumours registered the diagnosis had not been histologically verified (HV). For oesophageal cancer in males, an increase in both squamous cell carcinomas and adenocarcinomas was observed, with rates increasing from 3.5 to 4.4 per 100 000 for squamous cell type and 2.2 to 3.5 per 100 000 for the adenocarcinomas. These rises were accompanied by a decrease in the rate of non-HV tumours from 1.5 per 100 000 to 1.2. These patterns were also reflected in female rates, squamous cell cancers slowly increasing (from 2.5 per 100 000 in 1976 to 3.1 per 100 000 in 1989) with a smaller increase in the rate of adenocarcinomas (from 0.8 per 100 000 in 1976 to 1.1 per 100 000 in 1989). As in males, the increase in the incidence of such tumours was greater than the fall in the rate of unverified cancers (1976, 0.9 per 100 000; 1989, 0.7 per 100 000).

Over the entire period, 1975–90, 31% of gastric cancers were not HV, with a higher proportion unverified in the earlier years (1975–79, 39%; 1980–84, 30%; 1985–90, 24%) The proportion of gastric adenocarcinomas overall was 49% with a rise from 39% in 1975–79 to 60% in 1985–90. The rise in rates of adenocarcinomas was greater than the fall in non-HV tumours, with the former increasing by 21.3% and the latter falling by 15.7%. The proportion of HV tumours for which there is a non-specific morphology (e.g. neoplasms not otherwise specified, ICDO: 8000) has diminished.

Age-standardised rates for the five deprivation categories, varying from most deprived (category 5) to the most affluent (category 1), are presented for oesophageal and gastric cancer by sex in Figure 5a and b. A strong and statistically significant trend ($P = 0.005$) is seen for males associating oesophageal cancer with increasing levels of deprivation. In contrast, females do not appear to be at increasing risk with rising levels of deprivation ($P = 0.053$), although the highest rate is seen for the most deprived areas. Gastric cancer

**Figure 2** **a**, Age-specific incidence rates by mid-year of birth, oesophagus (ICD 150), 1961–90, males. **b**, Age-specific incidence rates by mid-year of birth, oesophagus (ICD 150), 1961–90, females.

**Figure 3** **a**, Age-specific incidence rates by mid-year of birth, stomach (ICD 151), 1961–90, males. **b**, Age-specific incidence rates by mid-year of birth, stomach (ICD 151), 1961–90, females.

**Figure 4** Age-standardised incidence rates of cancer of the oesophagus (ICD 150) by main tumour type, sex and mid-year of diagnosis, 1975–90.

**Figure 5** **a**, Age-standardised incidence rates by sex and deprivation category, 1975–90, oesophagus (ICD 150). **b**, Age-standardised incidence rates by sex and deprivation category, 1975–90, stomach (ICD 151). ■, Males; □, females.

exhibits similar trends for both sexes with steadily increasing rates as deprivation rises; the trends are both statistically significant (males, $P<0.001$; females, $P<0.001$).

### Discussion

In common with other countries throughout the world, stomach cancer is becoming less common in Scotland. In contrast, however, sizable increases have been recorded over the past 31 years in the incidence of oesophageal cancer in men and smaller but consistent increases in women. Time trends in cancer registration data may be explained by changes in registration quality. The process of cancer registration in Scotland has changed little over time, but it is possible that time trends in cancer incidence data may be explained by changes in registration quality. High levels of completeness of ascertainment may be indicated by a low proportion of cases registered from death certificates only (DCO) (Parkin *et al.*, 1992). Death certificates became a routine source of notification across Scotland in 1975, and the proportion of oesophagus DCO registrations fell from 5.2% in 1975–79 to 4.0% in 1985–89; for stomach cancer the comparable figures were 9.6% and 5.6% (Information and Statistics Division, unpublished data). These figures suggest improvements in registration efficiency over time, but are not of sufficient magnitude to explain entirely the changes in incidence reported in this paper. Increased ascertainment may also arise following the introduction of new diagnostic techniques. Endoscopic diagnosis of gastric cancer has become increasingly widespread and is likely to have resulted in at least small improvements in precision of registered information (Sedgwick *et al.*, 1991).

It has been noted that the decline in gastric cancer incidence in Scotland is less than the decline in mortality (Sedgwick *et al.*, 1991). A number of factors may account for this trend, including improved case ascertainment, increased survival and changes in, or differences between, registration and death certification coding practice. Five year survival rates in Scotland have improved marginally from 8.0% for those diagnosed in 1968–72 to 10.6% in 1983–87 (Black *et al.*, 1993). A similar disparity between incidence and mortality has been observed in Japan (Correa and Chen, 1994), which may be due to the registration of lesions which are biologically more benign than those recorded in the past (Coleman *et al.*, 1993). Compared with mortality figures, cancer registration data are likely to be more accurate, as the majority of cases are based on investigation of the tumour during life (Percy *et al.*, 1981). It is recommended in ICD-9 (World Health Organization, 1977) that tumours arising at the gastro-oesophageal junction should be coded to the stomach. Some misclassification of adenocarcinomas of the lower third of the oesophagus to the cardia of the stomach may occur in Scotland (Sharp *et al.*, 1993). Overall, interpretation of these data is problematic.

Oesophageal cancer rates are high in Scottish men compared with estimated average incidence rates in the European Union (EU). The increase in incidence rates in Scotland, however, is consistent with increases in oesophageal cancer mortality among men which have been noted in the EU (Macfarlane and Boyle, 1994). Rates of oesophageal cancer incidence in women in Scotland, in addition to being high relative to other geographical areas of the UK, are at least double those of other EU countries. The highest recorded incidence rate in the EU between 1983 and 1987 was from the East of Scotland Registry (4.9 per 100 000) (Parkin *et al.*, 1992). The increase in mortality among women in Scotland as well as other countries of the British Isles has not been occurring elsewhere in the EU (World Health Organization, 1992). When analysed by period of birth, it is evident that generally each successive birth cohort of males is experiencing increasing rates of oesophageal cancer at every age. In females, no such consistent pattern is evident, although in females over 55 years rates have been increasing over the 31 year period for which data have been collected. In some countries, for example Finland, an overall decreasing trend in females has been observed (Coleman *et al.*, 1993). In younger persons a slight increase in risk has been noted for males and females born after 1935 in Finland and in the USA (Connecticut) (Zheng *et al.*, 1992; Coleman *et al.*, 1993).

Recent interest has particularly focused on the region around the oesophagogastric junction. Despite falling rates of gastric cancer and considerable variability in the trends of oesophageal cancer worldwide, an increase in adenocarcinoma of the oesophagus occurring in the lower third and in the gastric cardia has been noted. Powell and McConkey (1990) have shown that in Birmingham (England), while the incidence rates of gastric cancer (all subsites) have fallen, the incidence rate of cardia tumours has increased in the same period, a change which cannot be explained by a decrease in the rate of subsite-unspecified tumours. Similarly, oesophageal adenocarcinoma, most commonly occurring around the oesophagogastric junction, has shown an increase over the same period, while squamous carcinoma has shown only a modest increase. Such changes have also been noted in the USA and Switzerland (Yang and Davis, 1988; Levi *et al.*, 1989; Blot *et al.*, 1991).

In Scotland the increase in oesophageal cancer in men has occurred in both squamous cell cancers (from 3.5 to 4.4 per 100 000 between 1976 and 1989) and adenocarcinomas (from 2.2 to 3.5 per 100 000 between 1976 and 1989). In females there has also been a steady increase in squamous cell cancers (from 2.5 to 3.1 per 100 000 between 1976 and 1989), while the incidence of adenocarcinomas has remained steady at around 0.85 per 100 000, and only more recently has a slight increase been noted (from 0.8 to 1.1 per 100 000 between 1976 and 1989). In neither sex are changes in the incidence of squamous cell carcinomas or adenocarcinomas entirely accounted for by changes in the proportion of histologically verified tumours. Increases in adenocarcinomas of the oesophagus may be interpreted as a likely increase in adenocarcinomas around the oesophagogastric junction owing to the difficulties in identifying the precise site of origin of tumours in this area. The Scottish data were not presented by subsite of either the oesophagus or the stomach, primarily because of the high proportion of cases with no subsite specified.

The reason for such increases in the incidence of squamous cell and adenocarcinomas of the oesophagus is unclear. Oesophageal cancer (predominantly squamous cell carcinomas) has been associated primarily with alcohol and tobacco consumption. Athough these two factors are highly correlated, they have been found to be independent risk factors (IARC, 1986, 1988) and their combined effects approximately multiplicative (Tuyns *et al.*, 1977). Additionally, increased risks of oesophageal cancer have been found in tobacco smokers who do not consume alcohol and alcohol drinkers who do not smoke tobacco (La Vecchia *et al.*, 1989).

Less information is available specifically on tumours around the oesophagogastric juntion. Adenocarcinoma of the oesophagus has previously been associated with tobacco smoking and alcohol drinking (Staszewski, 1974; Wang *et al.*, 1986; Wu-Williams *et al.*, 1990). Other studies have failed to find an effect, or have found an effect which is less than for squamous cell carcinomas (MacDonald and MacDonald, 1987; Li *et al.*, 1989; Gray *et al.*, 1992).

The relationship between tobacco smoking and alcoholic beverage consumption and gastric cancer is less clear: recent case–control studies do not show any relationship (Yu *et al.*, 1988; Buiatti *et al.*, 1989; La Vecchia *et al.*, 1989; Choi and Kahyo, 1991). Some studies have, however, previously found an association with smoking habits and alcohol consumption (Hu *et al.*, 1988; De Stefani *et al.*, 1990) or with smoking alone (Kono *et al.*, 1988).

Little information is available pertaining to the sub-site cardia, it does, however, exhibit a greater male–female ratio than other gastric sites – 2:9 for cardia compared with 1:4 for other stomach – with oesophagus also having a ratio of 2:9 (Powell and McConkey, 1990). In addition, the distribution of cases of gastric cardia with respect to social class and ethnic groups seems to be more closely related to oesophageal adenocarcinoma than to tumours occurring in the remainder of the stomach (Powell and McConkey, 1990; Blot *et al.*, 1991).

An area of recent interest has been a possible link between the presence of *Helicobacter pylori* (Hp) and the risk of gastric cancer. Indeed a correlation between risk and the 'prevalence' of Hp has been shown (Correa *et al.*, 1990) together with an increased prevalence in some groups at higher risk of gastric cancer, e.g. lower socioeconomic groups (Sitas *et al.*, 1991). Two recent case–control studies found a significantly increased risk of gastric cancer in those subjects who had been infected with Hp, excluding cases around the oesophagogastric junction (Nomura *et al.*, 1991; Parsonnet *et al.*, 1991).

Any hypothesis of reasons for the increase in tumours around the oesophagogastric junction is necessarily speculative given the lack of specific information. In contrast, tobacco and alcohol have been found to have an attributable risk in European countries of around 80% in men and 40% in women when considering all oesophageal tumours (Tuyns *et al.*, 1977; Negri *et al.*, 1992). Increases in the incidence of and mortality from intraoral cancers in Scotland, also strongly related to tobacco smoking and alcohol consumption, have likewise been found to be increasing (Macfarlane *et al.*, 1992). Such changes have occurred despite decreases in the prevalence of smoking and decreases in the incidence of and mortality from lung cancer in Scotland over the same period in men. In the UK the annual consumption of 100% alcohol per head of population has increased by 51.2% between 1968 and 1991. In Scotland a 650% increase in alcohol-related deaths over the same time period suggests that the rate of increase of consumption is greater in Scotland than in England and Wales (Scottish Council on Alcohol, 1994). It is likely that such an increase is contributing to the observed increasing rates. Overall, women consume less alcohol than men, but recent figures demonstrate a change in the pattern of consumption for women with increased frequency of drinking rather than more being drunk on each occasion as well as more women drinking over the recommended number of units per week (Thomas *et al.*, 1992). Rates of oesophageal cancer in Scottish women may reflect this alteration in the future.

Levels of deprivation can be assigned to geographical areas using indices derived from the national census data. Individuals with a cancer can then be allocated the index of the area in which they resided at diagnosis. This method of geographical correlation has shortcomings as it assumes that an individual will be representative of the area in which they live at a specific point in time, and this is obviously not always the case. Nonetheless, in the absence of accurate and complete measures of socioeconomic status which can be attached to individuals, such small area classifications are valuable in describing and explaining patterns of cancer incidence. This approach broadly describes associations with lifestyle factors and will provide crude evidence of underlying risk factors. The current study shows strong evidence of increasing risk of male oesophageal cancer in areas of high deprivation which may reflect higher levels of tobacco and alcohol consumption and poorer nutrition. Of interest is the less prominent effect for women, indicating that additional factors may be operational on the risk of disease for women. For men, the rates of both main tumour types increased with increasing deprivation. Squamous tumours showed a strong and consistent trend (50% higher in the most deprived areas than in the least deprived), while a lesser association was seen for adenocarcinomas. In women there was little evidence of a pattern for either tumour. Comparative data from elsewhere in the world are not apparent in published literature. Gastric cancer is clearly associated with high levels of deprivation likely to be accounted for by poor nutrition and a higher prevalence of Hp. Recent data show that variation in fruit and vegetable consumption across Scotland is linked to incidence of gastric cancer, with areas of low consumption displaying high rates of gastric cancer (SOHHD, 1993).

The descriptive analyses in the current paper show increasing rates of oesophageal cancer over time, in contrast to falling incidence for gastric cancer. An increase in adenocarcinomas of the oesophagus is not accounted for by the increasing proportion of HV tumours. This indicates the need for future epidemiological studies to focus on such tumours together with those occurring in the gastric cardia and to elucidate the factors associated with such a strong socioeconomic gradient of risk.

**Acknowledgements**

We wish to thank the Directors of the Regional Cancer Registries in Scotland and their staff for their work contributing to the national data set. We are grateful to Joan Roemmele for her assistance with producing our manuscript.

### References

ARMITAGE P AND BERRY G. (1971). *Statistical Methods in Medical Research*, 2nd ed. Blackwell Scientific Publications: Oxford.

BLACK RJ, SHARP L AND KENDRICK SW. (1993). *Trends in Cancer Survival in Scotland 1968–90.* Information and Statistics Division, NHS in Scotland: Edinburgh.

BLOT WJ, DEVESA SS, KNELLER RW AND FRAUMENI Jr JF. (1991). Rising incidence of adenocarcinoma of the oesophagus and gastric cardia. *J. Am. Med. Assoc.*, **265**, 1287–1289.

BOYLE P AND PARKIN DM. (1991). Statistical Methods for Registries In *Cancer Registration – Principles and Methods*. Machenman R, Muir CS and Skeet RG (eds). IARC Scientific Publications No. 95. IARC: Lyon.

BUIATTI E, PALLI D, DECARLI A, AMADORI D, AVELLINI C, BIANCHE S, BISERNI R, CIPRIANI F, COCCO P, GIACOSA J AND BLOT W. (1989). A case control study of gastric cancer and diet in Italy. *Int. J. Cancer*, **44**, 611–616.

CARSTAIRS V AND MORRIS R. (1991). *Deprivation and health in Scotland*. Aberdeen University Press: Aberdeen.

CHENG KK AND DAY NE. (1992). Oesophageal cancer in Britain. *Br. Med. J.*, **304**, 711.

CHOI S AND KAHYO H. (1991). Effect of cigarette smoking and alcohol consumption in etiology of cancers of the digestive tract. *Int. J. Cancer*, **49**, 381–386.

COLEMAN MP, ESTEVE J, DAMIECKI P, ARSLAN A AND RENARD H. (1993). *Trends in Cancer Incidence and Mortality*. IARC Scientific Publications, No. 121, pp. 1–806. IARC: Lyon.

CORREA P AND CHEN VW. (1994). Gastric cancer In *Trends in Cancer Incidence and Mortality, Cancer Surveys*, Vol. 20. Doll R, Fraumeni JF and Muir CS (eds). Cold Spring Harbour Laboratory Press: Cold Spring Harbor, NY.

CORREA P, FOX J, FONTHAM E, RUIZ B, LIN Y, ZAVALA D AND ZARAMA G. (1990). *Helicobacter pylori* and gastric carcinoma. *Cancer*, **66**, 2569–2574.

DE STEFANI E, MUNOX N, ESTEVE J, VASALLO A, VICTORA CG AND TEUCHMANN S. (1990). Mate drinking alcohol, tobacco diet and esophageal cancer in Uruguay. *Cancer Res.*, **50**, 426–431.

GRAY JR, COLDMAN AJ AND MACDONALD WC. (1992). Cigarettes and alcohol use in patients with adenocarcinoma of the gastric cardia or lower oesophagus. *Cancer*, **69**, 2227–2231.

HANSSON, L-E, SPAREN P AND NYREN O. (1993*a*). Increasing incidence of both major histological types of oesophageal carcinomas among men in Sweden. *Int. J. Cancer*, **54**, 402–407.

HANSSON, L-E, SPAREN P AND NYREN O. (1993*b*). Increasing incidence of carcinoma of the gastric cardia in Sweden from 1970 to 1985. *Br. J. Surg.*, **80**, 374–377.

HU J, ZHANG S, JIA E, WANG Q, LIU S, LIU Y AND CHENG Y. (1988). Diet and cancer of the stomach: a case-control study in China. *Int. J. Cancer*, **41**, 331–5.

INFORMATION AND STATISTICS DIVISION. *Indicators of the Accuracy of Data in the Scottish Cancer Registration Scheme, 1960–1989.* (unpublished data).

INTERNATIONAL AGENCY FOR RESEARCH ON CANCER (IARC). (1986). *Monographs on the Evaluation of the Carcinogenic Risk of Chemicals to Humans. Tobacco Smoking*, Vol. 38. IARC: Lyons.

INTERNATIONAL AGENCY FOR RESEARCH ON CANCER (IARC). (1988). *Monographs on the Evaluation of the Carcinogenic Risk of Chemicals to Humans. Alcohol Drinking*, Vol. 44. IARC: Lyon.

KONO S, IKEDA M, TOKUDOME S AND KURATSUNE M. (1988). A case control study of gastric cancer and diet in northern Kyushu, Japan. *Jpn J. Cancer Res.*, **79**, 1067–1074.

LA VECCHIA C AND NEGRI E. (1989). The role of alcohol in oesophageal cancer in non-smokers, and of tobacco in non-drinkers. *Int. J. Cancer*, **43**, 748–785.

LEVI F, MAISONNEUVE P, FILIBERI R, LA VECCHIA C AND BOYLE P. (1989). Cancer incidence and mortality in Europe. *Soz. Preventiv Med.*, **34**, (Suppl.), S1–S84.

LI J, ERSHOW AG, CHEN Z, WACHOLDER S, LI G, GUO W, LI B AND BLOT WJ. (1989). A case control study of cancer of the oesophagus and gastric cardia in linxian. *Int. J. Cancer*, **43**, 755–761.

MACDONALD WC AND MACDONALD JB. (1987). Adenocarcinoma of the esophagus and or gastric cancer. *Cancer*, **60**, 1094–1098.

MACFARLANE GJ AND BOYLE P. (1994). The epidemiology of oesophageal cancer in the United Kingdom and other European countries. *J. R. Soc. Med.*, **87**, 334–337.

MACFARLANE GJ, BOYLE P AND SCULLY C. (1992). Oral cancer in Scotland: changing incidence and mortality. *Br. Med. J.*, **305**, 1121–1123.

NEGRI E, LA VECCHIA C, FRANCESHI S, DECARLI A AND BRUZZI P. (1992). Attributal risks for oesophageal cancer in northern Italy. *Eur. J. Cancer*, **28a** (6 7), 1167–1171.

NOMURA A, STEMMERMANN GN, CHYOU P-H, KATO I, PEREZ-PEREZ GI AND BLASER MJ. (1991). *Helicobacter Pylori* Infection and gastric carcinoma among Japanese Americans in Hawaii. *N. Engl. J. Med.*, **325**, 1132–1136.

PARKIN DM AND MUIR CS. (1992). *Cancer Incidence in Five Continents*, Vol. VI. IARC Scientific Publications No. 120, pp. 45–173. IARC: Lyon.

PARSONNET J, FRIEDMAN GD, VANDERSTEEN DP, CHANG Y, VOLGLMAN JH, ORENTREICH N AND SIBLEY RK. (1991). *Helicobacter Pylori* infection and the risk of gastric carcinoma. *N. Engl. J. Med.*, **325**, 1127–1131.

PERCY C, STANEK E AND GLOECKLER L. (1981). Accuracy of cancer death certificates and its effect on cancer mortality statistics. *Am. J. Pub. Hlth.*, **71**, 242–250.

POWELL J AND MCCONKEY CC. (1990). Increasing incidence of adenocarcinoma of the gastric cardia and adjacent sites. *Br. J. Cancer*, **62**, 440–443.

REGISTRAR GENERAL SCOTLAND. (1960–1990). *Annual Reports 1960–1990.* HMSO: Edinburgh.

SCOTTISH COUNCIL ON ALCOHOL. (1994). *Alcohol Statistics*. SCA: Glasgow.

SCOTTISH HOME & HEALTH DEPARTMENT. (1993). *The Scottish Diet Report to the Chief Medical Officer*. SHHD: Edinburgh.

SEDGEWICK DM, AKOLIS JA AND MACINTYRE IMC. (1991). Gastric cancer in Scotland: changing epidemiology, unchanging workload. *Br. Med. J.*, **302**, 1305–1307.

SHARP L, BLACK RJ AND HARKNESS EF. (1993). *Cancer Registration Statistics in Scotland 1981–90.* Information and Statistics Division, NHS in Scotland: Edinburgh.

SITAS F, FORMAN D, YARNELL JWG, BURR ML AND ELWOOD P. (1991). Helicobacter Pylori infection rates in relation to age and social class in a population of Welsh men. *Gut*, **32**, 25–28.

STASZEWSKI J. (1974). Cancer of the upper alimentary tract and larynx in Poland and in the Polish-born Americans. *Br. J. Cancer*, **29**, 389–399.

THOMAS M, GODDARD E AND HUKMAN M. (1992). *General Household Survey*. OPCS, HMSO: London.

TUYNS AJ, PEQUIGNOT G AND ABBATUCCI JS. (1977). Le Cancer de L'oesophage en Ille-et-Vilaine en Fonction des Niveaux de Consummation D'alcool et de Tabac. Des Risques qui se Multiplient. *Bull. Cancer*, **64**, 45–60.

WANG HH, ANTONIOLOI DA AND GOLDMAN H. (1986). Comparative features of esophageal and gastric adenocarcinomas: recent changes in type and frequency. *Hum. Pathol.*, **17**, 482–487.

WORLD HEALTH ORGANIZATION (WHO). (1977). *Manual of the International Classification of Diseases, Injuries and Causes of Death*, 9th revision. HMSO: London.

WORLD HEALTH ORGANIZATION (WHO). (1992). *WHO Mortality Database*. WHO: Geneva.

WU-WILLIAMS AH, YU MC AND MACK TM. (1990). Lifestyle, workplace and stomach cancer by subsite in young men of Los Angeles County. *Cancer Res.*, **50**, 2569–2576.

YANG PC AND DAVIS S. (1988). Incidence of cancer of the esophagus in the US by histologic type. *Cancer*, **61**, 612–617.

YU MC, GARABRANT DH, PETERS JM AND MACK TM. (1988). Tobacco, alcohol, diet, occupation and carcinoma of the esophagus. *Cancer Res.*, **48**, 3843–3848.

ZHENG T, MAYNE S, HOLFORD T, BOYLE P, LIU W, CHEN Y, MADOR M AND FLANNERY J. (1992). The time trend and age period cohort: effects on incidence of oesophageal cancer in Connecticut. *Cancer Causes Control*, **3**, 481–492.